# *Angiostrongylus vasorum* in the eye: new case reports and a review of the literature

**DOI:** 10.1186/s13071-016-1440-6

**Published:** 2016-03-21

**Authors:** Vito Colella, Riccardo Paolo Lia, Johana Premont, Paul Gilmore, Mario Cervone, Maria Stefania Latrofa, Nunzio D’Anna, Diana Williams, Domenico Otranto

**Affiliations:** Dipartimento di Medicina Veterinaria, Università degli Studi di Bari, Valenzano, 70010 Italy; Eye-Vet Referrals, Apollo House, 41-43 Halton Station Road, Sutton Weaver, Nr Frodsham, Cheshire, WA73DN UK; Liverpool Veterinary Parasitology Diagnostics, University of Liverpool, IC2, Liverpool Science Park, 146 Brownlow Hill, Liverpool, L3 5RF UK; Small Animal Veterinary Clinic Paris III, Bl des Filles du Calvaire 17, Paris, 75003 France; Clinica per Animali Esotici, Centro Veterinario Specialistico, Rome, 00137 Italy

**Keywords:** *Angiostrongylus vasorum*, Lungworm, Ocular infection, Eye, Metastrongyloidea, Diagnosis, Snails

## Abstract

**Background:**

Nematodes of the genus *Angiostrongylus* are important causes of potentially life-threatening diseases in several animal species and humans. *Angiostrongylus vasorum* affects the right ventricle of the heart and the pulmonary arteries in dogs, red foxes and other carnivores. The diagnosis of canine angiostrongylosis may be challenging due to the wide spectrum of clinical signs. Ocular manifestations have been seldom reported but have serious implications for patients.

**Methods:**

The clinical history of three cases of infection with *A. vasorum* in dogs diagnosed in UK, France and Italy, was obtained from clinical records provided by the veterinary surgeons along with information on the diagnostic procedures and treatment. Nematodes collected from the eyes of infected dogs were morphologically identified to the species level and molecularly analysed by the amplification of the nuclear 18S rRNA gene.

**Results:**

On admission, the dogs were presented with various degrees of ocular discomfort and hyphema because of the presence of a motile object in the eye. The three patients had ocular surgery during which nematodes were removed and subsequently morphologically and molecularly identified as two adult males and one female of *A. vasorum*.

**Conclusions:**

Three new cases of canine ocular angiostrongylosis are reported along with a review of other published clinical cases to improve the diagnosis and provide clinical recommendation for this parasitic condition. In addition, the significance of migratory patterns of larvae inside the host body is discussed. Veterinary healthcare workers should include canine angiostrongylosis in the differential diagnosis of ocular diseases.

**Electronic supplementary material:**

The online version of this article (doi:10.1186/s13071-016-1440-6) contains supplementary material, which is available to authorized users.

## Background

Nematodes of the genus *Angiostrongylus* Kamensky, 1905 (Strongylida, Angiostrongylidae) are important because of their life-threatening potential in several animal species and humans [1]. Helminths within the superfamily Metastrongyloidea are usually known as “lungworms” because of their localisation in the lungs and associated blood vessels in the definitive host [2]. *Angiostrongylus* spp. develop in and are transmitted by gastropods (i.e. snails and slugs) which act as intermediate hosts [1, 3]. Amongst these parasites, *Angiostrongylus cantonensis* and *Angiostrongylus costaricensis* infect rodents and, occasionally, humans causing eosinophilic meningitis [4, 5] and abdominal angiostrongylosis [6], respectively. Dogs have been indicated as definitive hosts of *A. costaricensis*, suggesting that they may act as a reservoir host for this parasite in the domestic environment [7]. *Angiostrongylus vasorum* may cause severe clinical disease in dogs, red foxes and other carnivores, characterised by respiratory distress [8]. This parasitic infection has a patchy distribution in many parts of the world, including tropical, subtropical and temperate regions (i.e. Europe, Africa, North and South America) [9], and it is apparently expanding in new areas and around well-defined endemic foci [10]. The lack of international surveillance and difficulties in diagnosing *A. vasorum* impedes collection of data on its spread and global distribution [10–12]. Nevertheless, improved knowledge of parasite biology is required before drawing any definitive conclusion about the significance of the geographical expansion of *A. vasorum* [8, 9]. For instance, snail-to-snail transmission of infective third-stage larvae (L3) of *Aelurostrongylus abstrusus* (Strongylida, Angiostrongylidae) has been hypothesised as a key example for the spreading of nematodes associated with gastropod-borne diseases in endemic areas [13].

As with other metastrongylid nematodes, *A. vasorum* develops in snails and slugs from first-stage larvae (L1) to infective L3, in approximately 16 days [14]. In the canid definitive hosts, L3 undergo two moults in the abdominal lymph nodes and fifth stage larvae (L5) reach the right ventricle and pulmonary arteries, where they develop into dioecious adult nematodes [2, 15]. Gravid females lay eggs in the bloodstream that hatch in the respiratory system and L1 are passed out in the faeces [2, 15]. Canids may release L1 during their whole life, although the frequency of larval shedding varies over the year [8]. Along with this typical route of infection, frogs (*Rana temporaria*) may also act as intermediate and paratenic hosts of *A. vasorum* [16]. In addition, dogs can be experimentally infected with L3 shed in the environment from the snail *Biomphalaria glabrata* [17].

Clinical diagnosis of canine angiostrongylosis is challenging because of the wide spectrum of clinical signs and because subclinical infections also occur, leading to an underestimation of the true prevalence of infection [11]. Indeed, while respiratory signs are considered the main clinical presentation of the infection by *A. vasorum*, coagulative, cardiovascular and neurological disorders are also described [9]. The clinical picture can be further complicated by the fact that this condition may remain undiagnosed for months or even years [9]. Whilst respiratory and cardiac clinical signs are most commonly associated with *A. vasorum* infection, ocular manifestations have been seldom reported [18]. A better awareness of ocular angiostrongylosis will improve its diagnosis and treatment.

Here we report three new cases of canine ocular angiostrongylosis together with a review of previously published clinical cases.

## Methods

### Case presentation

Information on the clinical history of two cases of *A. vasorum* infection in dogs diagnosed in the UK and France (Cases 1 and 2, respectively) was obtained from clinical records provided by the veterinary practitioners along with information on the diagnostic procedures, treatment and outcome. A third case from Italy (Case 3) was also included [19]. In addition, we review cases reported in the international literature between 1913 and 2015, searching in Google Scholar and in the PubMed database the keywords “ocular angiostrongylosis”, “*Angiostrongylus vasorum* eye” and “*Angiostrongylus vasorum* ocular”.

### Morphological and molecular identification

Nematodes collected from the eyes of infected dogs were morphologically identified to the species level based on previous descriptions [20, 21]. In addition, identity of specimens extracted from Case 1 and 2 were confirmed by PCR. Briefly, genomic DNA from adult worms and L1 was extracted using a commercial kit (DNeasy Blood & Tissue Kit, Qiagen, GmbH, Hilden, Germany), in accordance with the manufacturers’ instructions. A nuclear 18S rRNA gene (~1700 bp) was amplified using the following primers (NC18SF1: 5'-AAA GAT TAA GCC ATG CA-3' and NC5BR: 5'- GCA GGT TCA CCT ACA GAT-3'). The amplicons were purified and sequenced using the Taq Dye Doxy Terminator Cycle Sequencing Kit (v.2, Applied Biosystems, Foster City, CA) in an automated sequencer (ABI-PRISM 377). Sequences were compared with those available in the GenBank database by Basic Local Alignment Search Tool (BLASTn http://blast.ncbi.nlm.nih.gov/Blast.cgi).

### Ethics statement

All medical procedures were carried with the owner’s approval. Nematodes were collected by veterinarians working in private clinics and sent to the Laboratory of Parasitology (University of Bari, Italy) for diagnostic purposes.

## Results

### Case 1

A 21-month-old female pug was referred to a veterinary opthalmologist with a one-day history of trauma to the right eye. The dog lived in a rural environment, on the edge of woods in the Greater Manchester area, in the vicinity of many animals (i.e. horses, sheep, pigs, chickens, and other dogs) and of a river, where the dog is usually walked. At the clinical visit, panuveitis with hyphema and fibrin deposition in the right eye were diagnosed; partial examination of the posterior segment showed no evidence of retinal detachment or posterior haemorrhages. Rebound tonometry revealed acute ocular hypertension with intraocular pressure of 30 mmHg in the right eye (20 mmHg in the left eye); however, a dazzle reflex was still present. No intraocular parasite was detected during the initial examination and there was no evidence of a systemic disease. The following day, the dog was sedated and a free floating worm was found in the anterior chamber of the right eye. The worm was aspirated *via* anterior chamber paracentesis using a 21 gauge needle and attached 2 ml syringe, placed in ethanol and subsequently morphologically and molecularly identified. An intracameral injection of 25 μg of tissue plasminogen activator and 0.1 mg of adrenaline was injected into the right anterior chamber to facilitate fibrinolysis, induce mydriasis and minimise further haemorrhage. Medical treatment consisted of chloramphenicol drops qid, brinzolamide drops qid, prednisolone acetate drops qid, nepafenac drops qid, cephalexin 15 mg/kg bid and prednisolone 0.5 mg/kg SID *per os*. Milbemycin oxime 12.5 mg combined with praziquantel 125 mg (Milbemax®, Novartis Animal Health) was prescribed once a week for 4 weeks, then once monthly long term.

**Fig. 1:**
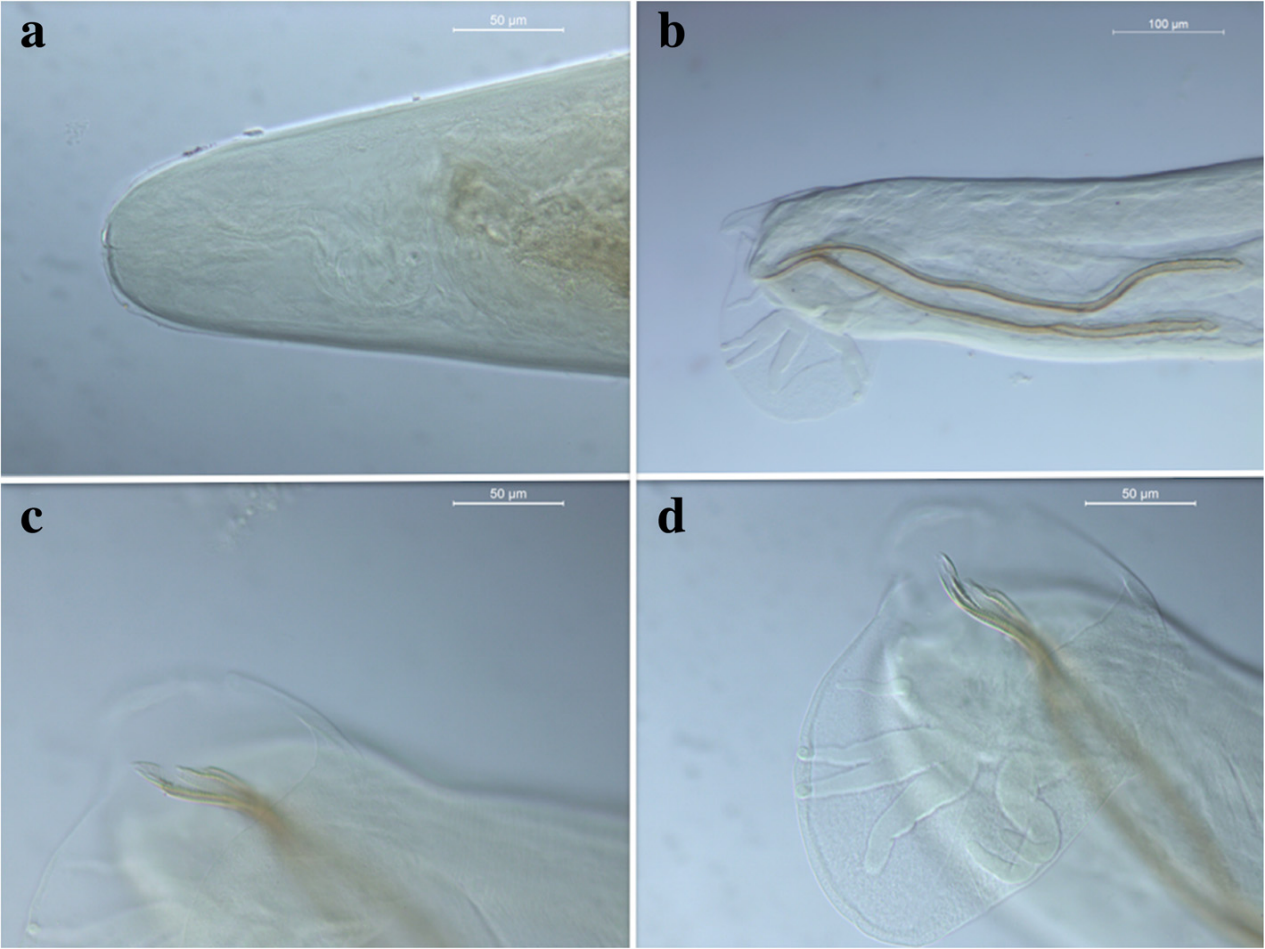
Case 1. Cephalic extremity of *Angiostrongylus vasorum* (**a**); Short copulatory bursa featured by well-developed bursal rays (**b**); Strongly sclerotised spicules with a thin membrane (**c**); Spicules measured 0.456 mm and 0.459 mm in length (**d**)

The parasite was broken at the level of the anterior extremity measuring the two main pieces 2.13 mm and 4.51 mm in length; the width was 0.118, 0.28 and 0.182 mm at the anterior, middle and posterior ends, respectively. The nematode had a moderately dilated cuticle at the cephalic extremity, with a small buccal aperture (Fig. 1a). The oesophagus measured 0.219 mm in length, and the excretory pore was located at 0.410 mm from oesophagus. The posterior extremity was ventrally curved, with a small copulatory bursa with well-developed bursal rays (Fig. 1b). Spicules appeared yellowish and strongly sclerotised and transversally striated and measured 0.456 mm and 0.459 mm in length (Fig. 1c, d). A slightly sclerotised ellipsoidal gubernaculum was located close to the terminal end of the spicules. The nematode was identified as a male of *A. vasorum*. Further, the 18S rDNA sequence obtained from the adult nematode displayed 100 % identity to the nucleotide sequence of *A. vasorum* [GenBank: AJ920365].

A follow-up 2 weeks later, revealed complete resolution of the anterior uveitis and hyphema. Two 1 mm white opacities and one small resorbing hemorrhage were detected retrolenticularly in the anterior vitreous. Further examinations, investigations and treatment were declined by the owners. A follow-up by telephone was performed 20 months later and the owner reported no further evidence of ocular or systemic clinical signs.

### Case 2

**Fig. 2:**
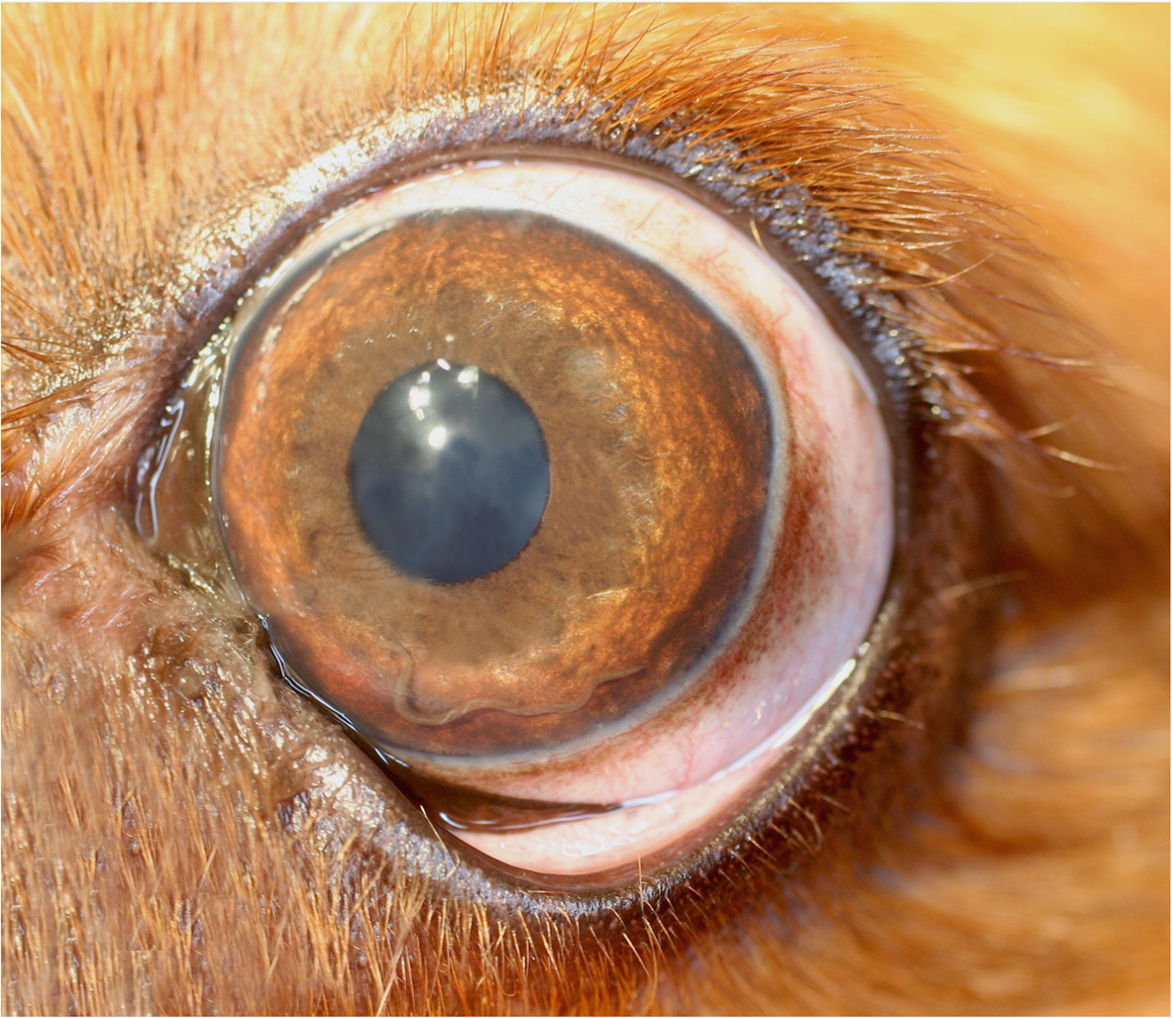
Case 2. *Angiostrongylus vasorum* in the anterior chamber of the left eye of a dog

A 2-year-old male Cavalier King Charles Spaniel dog was referred to a private veterinary clinic in Paris (France) due to persistent blepharospasm and epiphora. The dog that lived in the city centre was walked in a forest nearby. On clinical ophthalmological examination the dog showed prolapse of the nictitating membrane, photophobia on the left eye, iris hyperaemia and corneal oedema. The intraocular pressure was 14 mmHg in the left eye and 16 mmHg in the right eye, the fluorescein test was negative and the Schirmer test showed a slightly increased lacrymation. A thread-like organism was noticed in the anterior chamber of the left eye (Fig. 2), which was very motile under light stimuli. An additional movie file clearly shows this in more detail (see Additional file 1).

Removal of the parasite was performed by anterior chamber paracentesis and the nematode was morphologically and molecularly processed. The specimen was identified as an unfertilised *A. vasorum* female. Briefly, the female nematode measured 16 mm in length and 0.2 mm in width; genital ducts were coiled around the reddish intestine, which appeared visible throughout the cuticle. However, the nematode was damaged and further morphological features were not evaluated, with the exception of the uteri, which lacked first-stage larvae, and the vulva. Therefore, the dog was subjected to coprological examination for the detection of L1 using the Baermann technique. The 18S rDNA sequences obtained from both L1 collected from the faeces and the female nematode displayed 100 % identity to the nucleotide sequence of *A. vasorum* [GenBank: AJ920365]. The dog lacked any signs of respiratory infection, both previously and during the observation period. The animal was treated with fenbendazol 25 mg/Kg *per os* SID for 3 weeks associated with prednisolone 0.2 mg/Kg.

### Case 3

**Fig. 3:**
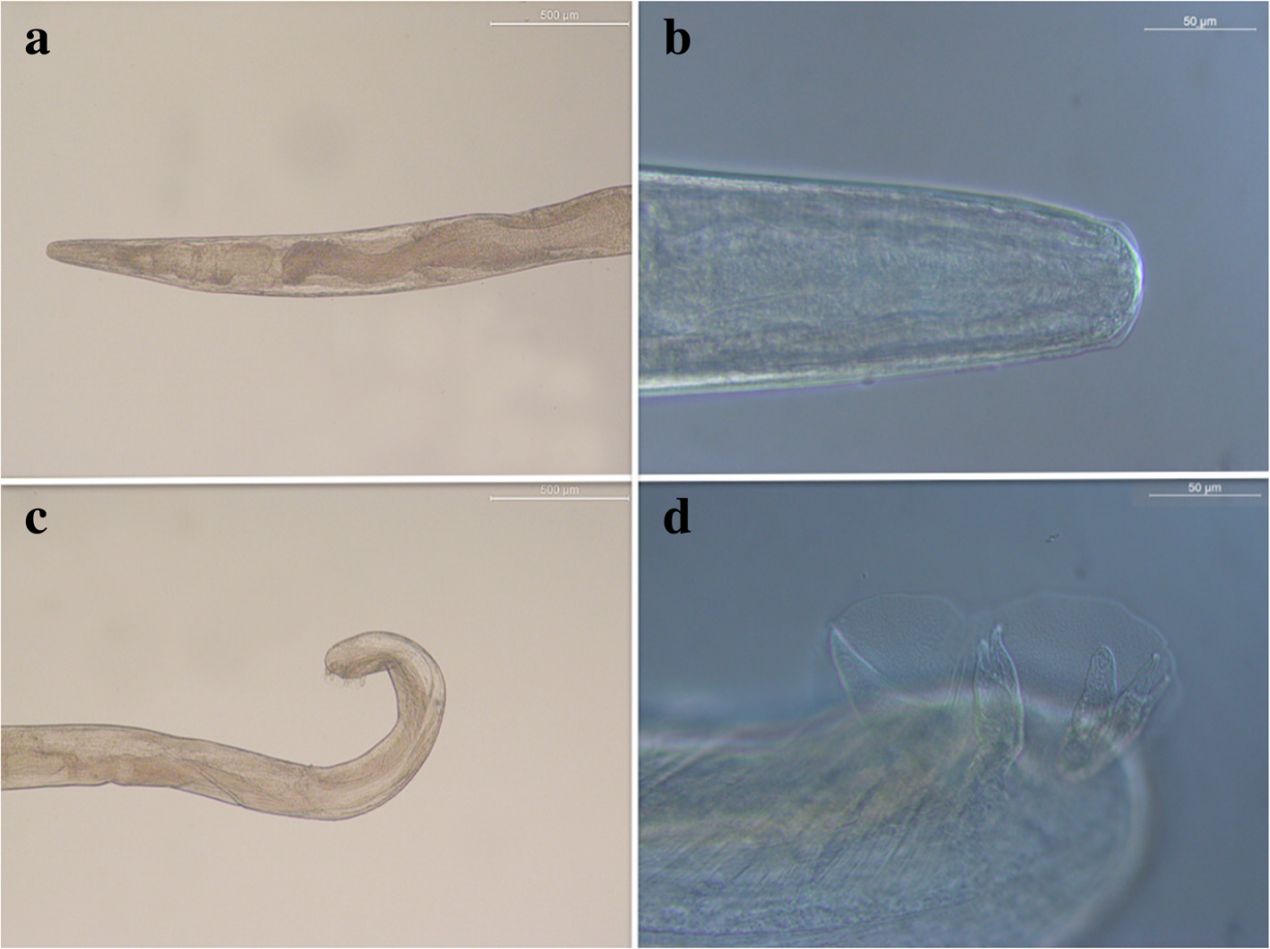
Case 3. Smooth cuticle and slender body of *Angiostrongylus vasorum* (**a**); Anterior extremity (**b**); Posterior extremity (**c**); Posterior extremity at higher magnification showing a short copulatory bursa (**d**)

A 5-month-old male mixed breed dog was referred to a private veterinary clinic in Rome (Italy) for sudden visual loss. At the admission, the clinical ophthalmological examination revealed corneal oedema and episcleral congestion in the right eye. Examination of the anterior chamber showed diffuse hyphema and complete examination of the posterior segment could not be performed. Clinical diagnosis of anterior uveitis in the right eye was made. Ultrasound of the right eye displayed a blood clot in the anterior chamber. CBC, biochemistry and electrolytes were within the limits. Topical dexamethasone 0,2 %, four times a day, was administered for 2 weeks. Fifteen days following the first examination, the hyphema disappeared and the presence of a free-swimming nematode in the anterior chamber was noticed. Subsequently the dog was anesthetised and the eye was clipped and prepared for surgery. Under surgical microscope the cornea was incised, the anterior chamber was flushed with balance saline solution (BSS) and the nematode was collected and morphologically identified. Briefly, the parasite presented a smooth cuticle and a slender body with a small buccal aperture (Fig. 3a), and measured 10.5 mm in length and 0.29 mm in width at the middle portion, the anterior and posterior extremities were 0.109 and 0.176 mm in width, respectively (Fig. 3b, c). The oesophagus was 0.219 mm long. The posterior end was ventrally curved, with a short copulatory bursa (Fig. 3d). The subequal spicules measured 0.404 mm and 0.388 mm in length. The nematode was morphologically identified as a male of *A. vasorum*. Unfortunately, due to inadequate preservation of the specimen, the extraction and amplification of genomic DNA was unsuccessful. Stool samples were taken to look for L1 with Baermann technique and tested negative. The animal recovered without complications after the surgery. Topical and systemic antibiotics and steroids were administered for the next 3 weeks. The dog was re-examined at 2 and 6 months post-operatively and ocular examinations were within normal limits.

## Discussion

Three cases of ocular infection with *A. vasorum* in dogs are described along with clinical presentations and diagnostic procedures. This information is vital to help our understanding of the clinico-pathological picture of canine ocular angiostrongylosis. Several reports of aberrant migration of *A. vasorum* are described in the scientific literature, with adult worms recovered from left ventricle, pericardial sac, urinary bladder and femoral artery [22–24], and L1 from brain, kidney, liver, muscle, stomach, pancreas and spleen of infected dogs [22, 23, 25]. However, to date, ocular migration of *A. vasorum* has rarely been described. Cases of canine ocular angiostrongylosis have been reported in France [26–28], United Kingdom [18, 29], Denmark [30] and Canada [25] (Table 1), although many infections may be unreported or inadequately described. For instance, in a series of six cases of ocular parasitosis described by [18], dogs presented clinical signs strongly suggestive of *A. vasorum* infection but the aetiology of these conditions was not confirmed by any morphological or molecular identification. Our findings suggest that *A. vasorum* infection should be considered if ophthalmological examination reveals a nematode in the eye even in the absence of other typical clinical signs and L1 are not detected in faeces [11, 18, 28]. In Case 2 as well as in the report by Rosenlund et al*.* [30], despite the absence of any cardiopulmonary sign, L1 were found in the faeces only after the diagnosis of ocular angiostrongylosis. Conversely, in Case 3 and in the report by Payen [28], L1 were not detected in the faeces. Although in the case described by Payen [28] the dog was presented with overt cardiorespiratory clinical signs, the diagnosis of *A. vasorum* infection was based on the identification of the nematode in the anterior chamber of the eye. As shown in Case 3 and by King et al*.* [29], dogs were presented with varying degrees of ocular discomfort, and only upon re-examination six to 15 days later, was the presence of a free-swimming nematode detected. This emphasises the importance of clinical follow-up in order to eventually diagnose ocular infection with *A. vasorum*.

**Table 1:** Review of cases described in the literature of canine ocular angiostrongylosis, along with data on the location, clinical presentation, diagnosis and anthelmintic treatment

Age, sex and breed	Location	Clinical presentation	Diagnosis	Anthelmintic treatment	Reference
8-month-old; n/a; Cavalier King; Charles Spaniel	France	Cough, uveitis, and presence of a motile worm in the anterior chamber of the eye	Faecal and broncho-alveolar washing examination negative for *A. vasorum* larvae.	Fenbendazole-based treatment for 2 weeks	[28]
			One immature *A. vasorum* female extracted from the eye		
14-month-old; female; Cocker Spaniel	Denmark	Epiphora, circumcorneal and conjunctival injection of the episcleral blood vessels, blepharospasm, prolapse of the nictitating membrane miotic and swollen iris. Presence of a motile worm in the anterior chamber of the eye	Numerous *A. vasorum* L1 found in the faeces. One immature *A. vasorum* female extracted from the right eye.	Levamisole injections 10/mg kg for 3 days	[30]
3-year-old; female; Cavalier King Charles Spaniel	UK	Otitis interna, head tilt, submaxillary lymph node enlargement. Presence of a motile worm in the anterior chamber of the eye on a one-day follow up visit. The dog died from a sudden attack of acute respiratory distress	Numerous *A. vasorum* adults in the right ventricle and pulmonary artery and one adult in the anterior chamber detected at post-mortem examination	n/a	[29]
2-year-old; female; Staffordshire Bull Terrier	UK	Post-inflammatory retinal degeneration, episcleral hyperaemia and vitreous herniation. Presence of a motile worm in the anterior chamber of the left eye	*A. vasorum* L1 found in the faeces. No morphological or molecular identification of the nematode found in the left eye	Fenbendazole 50 mg/kg for 10 days	[18]
20-month-old; male; Miniature Dachshund	Canada	Chronic diarrhoea and coughing. Ataxia, depression, and impaired vision. Multiple retinal haemorrhages, iris congestion. The dog was euthanised due to the progression of nervous and ocular diseases	Numerous *A. vasorum* adults recovered from pulmonary artery and disseminated larval infection including both eyes, kidneys, brain, spinal cord, bronchial lymph nodes, heart, intestine and pancreas, detected at post-mortem examination	n/a	[25]

Other nematodes have been identified in the eyes of dogs, notably fourth-stage larvae of *Dirofilaria immitis* (Spirurida, Onchocercidae) which have been shown to migrate in the anterior chamber and vitreous body of the eye [31, 32]. In addition, adult *Onchocerca lupi* (Spirurida, Onchocercidae) usually localise in the subconjunctival granulomas and/or in the retrobulbar space of the eye of infected dogs [33, 34], and recently intraocular onchocercosis has also been described in a patient suffering from anterior uveitis [35]. The availability of a diagnostic test for the detection of circulating *A. vasorum* antigens in dogs (IDEXX Angio Detect™) [36] has provided a further tool to assist a definitive parasitological diagnosis. Interestingly, all dogs suffering from canine ocular angiostrongylosis were under the 3 years of age (i.e. 5 months to 3 years), and of the few reports now available in literature, three [28, 29] and in Case 2, involved Cavalier King Charles Spaniel dogs. However, additional epidemiological data are needed for a clear assessment of risk factors (e.g. breed and age) related to the occurrence of canine ocular angiostrongylosis.

How *A. vasorum* larvae reach the ocular tissues of dogs is not clear, although migration may take place by penetration of the corneal surface (cranial-hypodermis route), the surface of the brain and the optic foramen (intracranial-optic foramen route) or through a fibrin sac in the anterior chamber of the eye (corneal route), in a similar manner to those reported for *D. immitis* [37]. The hyphema described in Case 1 and 3 has been associated with the infection as a likely consequence of coagulative disorder or a traumatic effect of the adult nematode on the ocular tissues. This could explain the finding of the adult worm in the anterior chamber only at the second examination. Nevertheless, further clinico-parasitological research is necessary to ascertain the routes of migration and the pathogenesis of ocular angiostrongylosis in dogs. For instance, for many zoonotic helminths affecting the eyes, the parasitic localisation in the ocular tissues or the immune reaction elicited by adults or larval stages in the host are of primary importance in the appearence of overt clinical signs [38].

Milbemycin oxime combined with praziquantel once a week for 4 weeks and fenbendazole (25 mg/kg) for 3 weeks were used to treat *A. vasorum* infection in Case 1 and 2, respectively. Several pharmaceutical options are now available and highly efficacious in treating canine angiostrongylosis, including moxidectin/imidacloprid spot on solution (Advocate®, Bayer Animal Health) with a single monthly application [39, 40], and milbemycin oxime in combination with praziquantel (Milbemax®, Novartis Animal Health) administered weekly for 4 weeks [41].

## Conclusion

Aberrant migration of nematodes increases the complexity of the clinico-pathological picture of canine angiostrongylosis, thus an enhanced awareness of clinical conditions caused by *A. vasorum* is imperative for the diagnosis and treatment of this infection. Recognition of the importance of alternative migratory routes of *A. vasorum* in dogs will improve our current understanding of the diagnosis and clinical follow-up of this parasitic condition. For example, veterinary healthcare workers should include canine angiostrongylosis in the differential diagnosis of ocular diseases. Finally, in a review of 484 cases of human eosinophilic meningitis caused by *A. cantonensis*, 47 patients (9.7 %) suffered from ocular disease [42]. In addition, at least 35 patients were further diagnosed with human ocular angiostrongylosis [43]. Therefore, a better appreciation of ocular angiostrongylosis in dogs may assist in our understanding of human ocular angiostrongylosis.

## Additional file

Additional file 1: Case 2. *Angiostrongylus vasorum* in the anterior chamber of the left eye of a 2-year-old male Cavalier King Charles Spaniel dog. (MOV 1003 kb)
